# Stable Field Emission from Vertically Oriented SiC Nanoarrays

**DOI:** 10.3390/nano11113025

**Published:** 2021-11-11

**Authors:** Jianfeng Xiao, Jiuzhou Zhao, Guanjiang Liu, Mattew Thomas Cole, Shenghan Zhou, Ke Chen, Xinchuan Liu, Zhenjun Li, Chi Li, Qing Dai

**Affiliations:** 1 Henan Institute of Advanced Technology, Zhengzhou University, Zhengzhou 450001, China; xiaojf2020@nanoctr.cn (J.X.); daiq@nanoctr.cn (Q.D.); 2 CAS Key Laboratory of Nanophotonic Materials and Devices, CAS Key Laboratory of Standardization and Measurement for Nanotechnology, CAS Center for Excellence in Nanoscience, National Center for Nanoscience and Technology, Beijing 100190, China; zhaojz2020@nanoctr.cn (J.Z.); liugj20101120@163.com (G.L.); zhoushenghan@nanoctr.cn (S.Z.); chenke@nanoctr.cn (K.C.); liuxc2019@nanoctr.cn (X.L.); 3 Department of Electronic and Electrical Engineering, University of Bath, Bath BA2 7AY, UK; mtc47@bath.ac.uk; 4 Center of Materials Science and Optoelectronics Engineering, University of Chinese Academy of Sciences, Beijing 100049, China; 5 GBA Research Innovation Institute for Nanotechnology, Guangzhou 510700, China

**Keywords:** silicon carbide, one-dimensional nanomaterials, nanoarrays, field emission

## Abstract

Silicon carbide (SiC) nanostructure is a type of promising field emitter due to high breakdown field strength, high thermal conductivity, low electron affinity, and high electron mobility. However, the fabrication of the SiC nanotips array is difficult due to its chemical inertness. Here we report a simple, industry-familiar reactive ion etching to fabricate well-aligned, vertically orientated SiC nanoarrays on 4H-SiC wafers. The as-synthesized nanoarrays had tapered base angles >60°, and were vertically oriented with a high packing density >10^7^ mm^−2^ and high-aspect ratios of approximately 35. As a result of its high geometry uniformity—5% length variation and 10% diameter variation, the field emitter array showed typical turn-on fields of 4.3 V μm^−1^ and a high field-enhancement factor of ~1260. The 8 h current emission stability displayed a mean current fluctuation of 1.9 ± 1%, revealing excellent current emission stability. The as-synthesized emitters demonstrate competitive emission performance that highlights their potential in a variety of vacuum electronics applications. This study provides a new route to realizing scalable field electron emitter production.

## 1. Introduction

One-dimensional (1D) SiC nanostructures have been widely considered comparable to carbon nanotubes (CNTs) [1] as being near ideal candidates for field-emission (FE) applications given their low electron affinity, high electron mobility, and high breakdown field strength, outstanding chemical and physical stability, and high thermal conductivity [2,3,4]. Such chemical and physical properties make SiC suitable for use in high-power, high-voltage, and high-temperature, and otherwise aggressive environments [5]. Indeed, to date, the turn-on fields (E_to_, generally defined as the electric field to generate a current density of 10 μA/cm^2^) of 1D SiC nanostructures is commonly several V/µm [6,7,8,9], and although higher than that of CNTs, it does support the highly stable emission of very high voltage and current densities highlighting the material’s potential as a novel high power FE material system [10,11,12].

Typical SiC 1D nanostructures, including nanowires [13], nanobelts [7], nanorods [14], and nanoneedles [15], have been realized via a variety of different fabrication methods [4,5,16], which are broadly classified, either via direct synthesis from carbon/silicon sources or via the processing of SiC substrates [8,17]. Although the SiC 1D nanostructures fabricated in these ways have showed excellent FE properties [8,18,19,20], most are produced in relatively harsh synthesis environments, often requiring very high, CMOS-incompatible processing temperatures and toxic gaseous precursors. It remains a significant engineering challenge to fabricate large-area 1D SiC nanostructures in a cost-effective manner using simple, widely accessible laboratory equipment under nominally safe process conditions.

Dry etching is a well-established, mature technology that has been widely used in the silicone industry for more than four decades [21]. Dry etching has, as a result, become one of the central processing steps in the fabrication of many SiC nanostructures on SiC wafers [22,23,24,25]. In the present work, vertically oriented SiC nanoarrays have been successfully synthesized under controlled pressure, etching gas ratio, and etching time in a commercially available reactive ion etching system. Our field electron emission measurements suggest an E_to_ of the as-synthesized SiC nanoarrays as low as 4.3 V/μm, and current emission of ~5 mA/cm^2^, making them competitive with other established electron emission materials including CNT [26], Si [27], ZnO [28], and diamond [29]. Current emission stability tests demonstrated time fluctuations of 1.9 ± 1% over 8 h. These findings outline the potential use of the fabricated SiC nanoarrays in new FE systems that require high lifetime and high intrinsic temporal stability, such as traveling wave tubes [30], X-ray resources [31,32] and flat panel displays [5].

## 2. Materials and Methods

**Figure 1:**
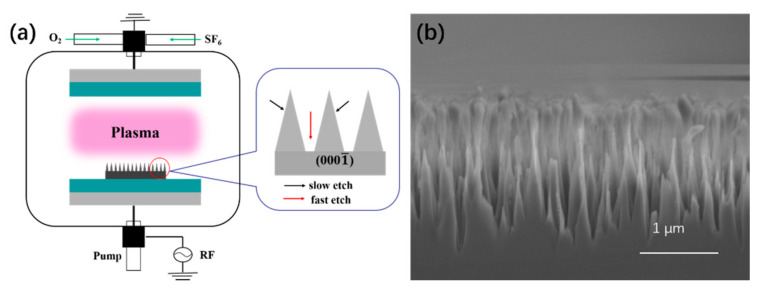
(**a**) Schematic diagram of RIE etching 4H-SiC wafer substrate and (**b**) cross-section of scanning electron microscope (SEM) image after RIE etching for 20 min.

The vertically oriented SiC arrays were fabricated on commercially available 4H-SiC substrates (TanKeBlue Semiconductor Co. Ltd, Beijing, China) via reactive ion etching ( ETCHLAB 200, SENTECH Instruments GmbH, Berlin, Germany). The fabrication process is outlined in Figure 1a. In a typical process, a 4-inch 4H n-type SiC ((0001), 0.02 Ω cm ) single-crystal substrate was diamond scribed into 1 × 1 cm^2^ samples. These were then cleaned by ultrasonication in acetone for 15 min, followed by rinsing in isopropanol and deionized water prior to a final clean in HF and ethanol (HF (40%): C_2_H_5_OH (99%) = 1:1) for 3 min to remove any surface oxides prior to RIE. RIE on the C-face (000$\bar{1}$) was conducted at 200 W at 5 Pa under 16 sccm SF_6_ (Beijing Huanyu Jinghui Jingcheng Gas Technology Co., Ltd, Beijing, China, 99.999%) and 4 sccm O_2_ (99.999%, Beijing Huanyu Jinghui Jingcheng Gas Technology Co., Ltd, Beijing, China), with the chamber being ashed with O_2_ plasma (200 W, 5 Pa, 10 min) prior to sample etching. After RIE, the etched wafers were immersed in HF and ethanol (HF (40%): C_2_H_5_OH (99%) = 1:1) for 3 min for a second time so as to remove newly grown surface oxides. To electrically contact the produced nanoarrays, an ohmic contact was formed on the substrate backside with Ni (100 nm) deposited by electron beam evaporation (OHMIKER-50B, Cello Technology Co., Ltd, Hsinchu, China) annealed at 950 °C at <10 Pa under 100 sccm Ar (99.999%, Chengdu Jinghao Chemical Products Co., Ltd, Chengdu, China) [33].

**Figure A1:**
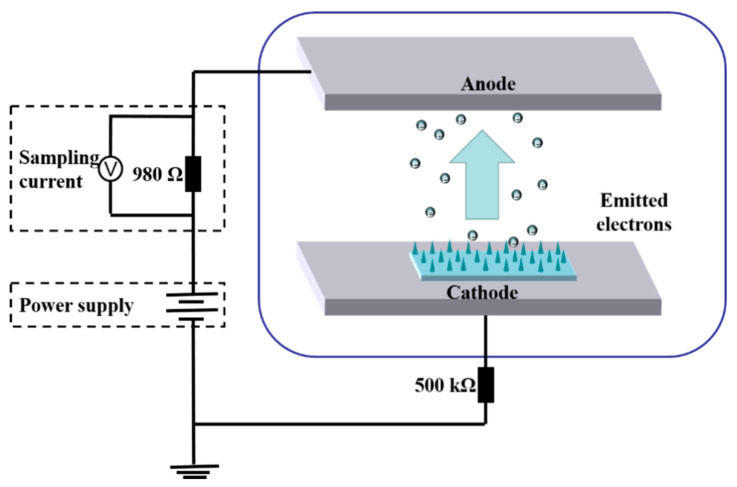
Schematic diagram of field-emission measurement.

FE measurements, the scheme of which is shown in Figure A1, were conducted in a custom-built, continuously turbo-pumped vacuum system operating at a base pressure of <5 × 10^−6^ Pa at room temperature. The SiC wafer covered with 0.9 cm^2^ of nanoarrays was fixed on the sample table using insulating tape with the anode–cathode separation distance fixed at 300 μm, and the SiC nanoarrays were facing towards an adjacent electrode. An adjustable positive voltage (0~10 kV) was applied to a polished steel counter anode using a power supply (DW-P103-20, Tianjin Dongwen High Voltage Power Supply Co., Ltd, Tianjin, China). A 980 Ω resistor was used to measure the emission current, with a 500 kΩ current limiting resistor connected in series to protect the power supply. Four samples fabricated with different etching times were measured in each FE measurement.

The obtained samples were characterized by scanning electron microscopy (FESEM, Hitachi-SU8220, Hitachi, Tokyo, Japan), and an energy-dispersive X-ray spectrometer (EDS) equipped high-resolution transmission electron microscopy (HR-TEM, Tecnai G2 20 S-TWIN, Bionand, Málaga, Spain). The emitting surface work function (WF) was measured by ultra-violet X-ray photoelectron spectroscopy (UPS) using a He I discharge lamp (hν = 21.2 eV), with the WF being extracted from the secondary electron cut-off under a sample bias of −10 V. The position of the Fermi level was calibrated by measuring the Fermi edge of a sputter-cleaned gold sample.

## 3. Results and Discussion

**Figure 2:**
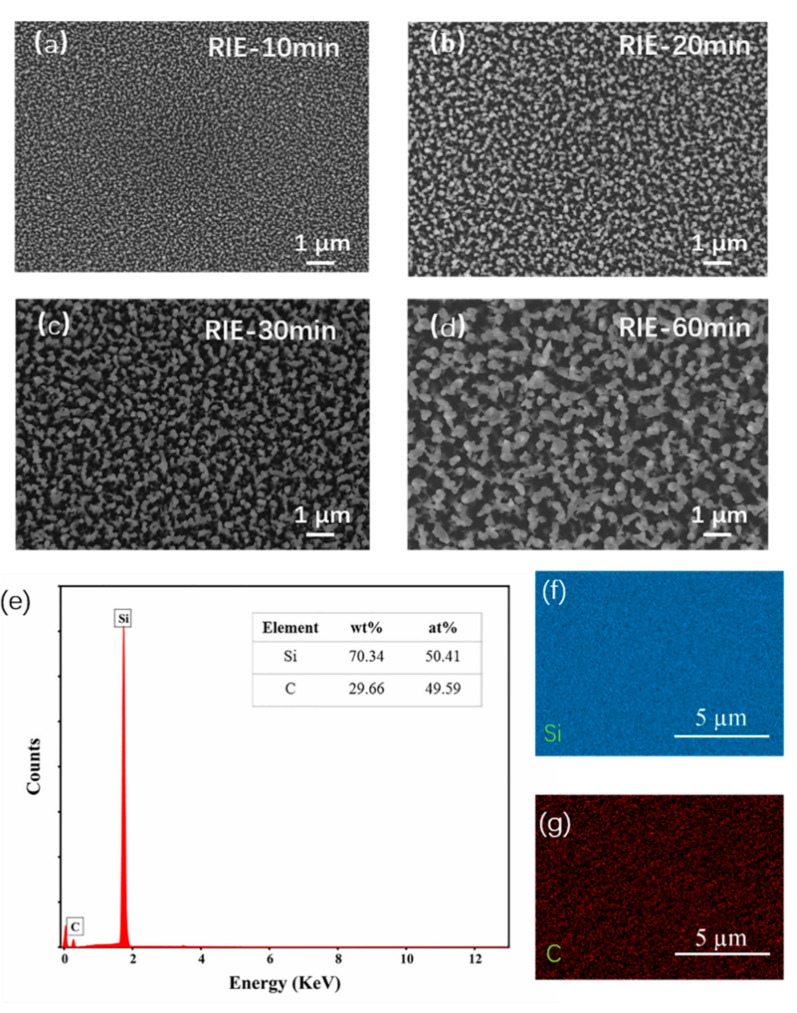
SEM images (plan view) of SiC nanoarrays prepared by RIE etching for (**a**) 10 min, (**b**) 20 min, (**c**) 30 min, and (**d**) 60 min, respectively (scale bar: 1 µm). (**e**) Energy-dispersive X-ray (EDX) spectrum of nanoarrays etched for 20 min, the inset is the proportion of the chemical compositions. (**f**,**g**) EDX elemental mapping.

**Figure A2:**
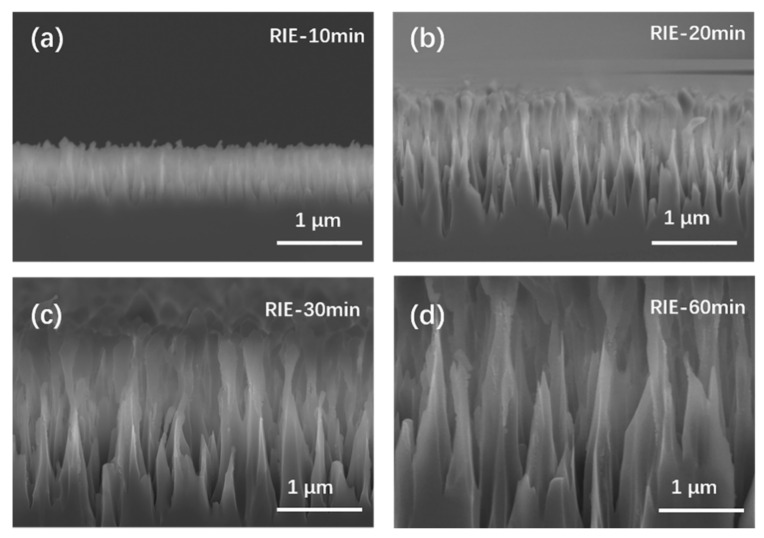
Cross-sectional SEM images of the silicon carbide nanoarray after RIE etching for (**a**) 10, (**b**) 20, (**c**) 30, and (**d**) 60 min, respectively. Scale bar: 1 μm.

These nanoarrays were formed by RIE etching on 1 × 1 cm^2^ samples, without masking, to allow samples to be mounted in the size-limited FE measurement system. The mechanism of forming such vertically-oriented large-scale SiC nanostructures was outlined by Liu et al [34]. Figure 1a depicts the RIE setup. Figure 1b depicts a cross-sectional SEM image of a 20-min RIE etched sample. Due to surface energy minimization, the etch rate is notably faster on the (000$\bar{1}$) C face (approximately 75 nm/min) than on other etch-active planes (<10 nm/min). This results in the formation of spike-like nanostructures. Figure 2a–d show typical top-view SEM images of the as-synthesized SiC nanoarrays, fabricated by the outlined RIE etching of 4H-SiC following 10 min, 20 min, 30min, 60 min etching. The vertically oriented and high packing density cone-like SiC nanoarrays have a random spatial distribution with an approximate tip density of 10^6^–10^8^ mm^−2^. We note that as the etching time increases the radius of the tips gradually becomes larger at a rate of ~5 nm/s. Nanostructures have a vertically-oriented configuration, generally with a length of 0.75–4.35 µm and a base/tip diameters of 200–1000 nm/30–300 nm (as shown in Figure A2), with their geometry being comparable to commercially incumbent emitters, highlighting their potential use as large-area field emitters.

**Figure 3:**
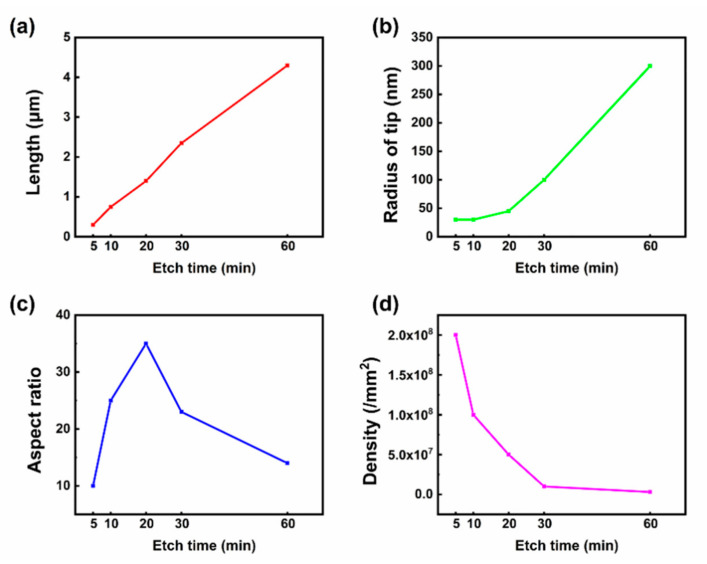
Plots of (**a**) length and (**b**) radius at the tip of a single nanocone under different etching times. (**c**) Plots of the aspect ratio of a single nanocone under different etching times. (**d**) Plots of density and etching time dependence.

In order to explore the effect on the nanoarray geometry as a function of the etching time, we calculated the density, the length of a single nanocone, the tip radius, and the aspect ratio (defined as the ratio of length to tip radius) and plotted them as shown in Figure 3. As the etching time increases, the density of the SiC nanoarrays reduces (Figure 3d), the length of nanostructures increases (Figure 3a), the radius of the tip increases (Figure 3b), with the aspect ratio reaching a maximum at an etch time of 20 min (Figure 3c). Etching over extended time frames (>30 min) appears to homogenize the surface, with many of the smaller/finer, yet often very FE active tips being removed, resulting in more uniform deep etches.

In order to engineer high-performance FE emission systems, it is essential to understand the chemical composition of the emitters’ uppermost electron-emitting surface. In order to examine the chemical compositions of the SiC nanoarrays, EDS mapping was conducted over sample areas of up to ~120 µm^2^. A typical EDS spectrum and map are shown in Figure 2e. The emitting surface was found to be extremely chemically uniform. They consisted principally, as expected, of Si (50.4 at%) and C (49.6 at%), in a near 1:1 ratio. Very little sample oxidation was noted (<0.1 at%), evidencing the efficacy of the post-RIE HF oxide etch. These findings suggest that the RIE dry etching does not introduce additional chemical impurities to the SiC nanoarray during processing.

**Figure 4:**
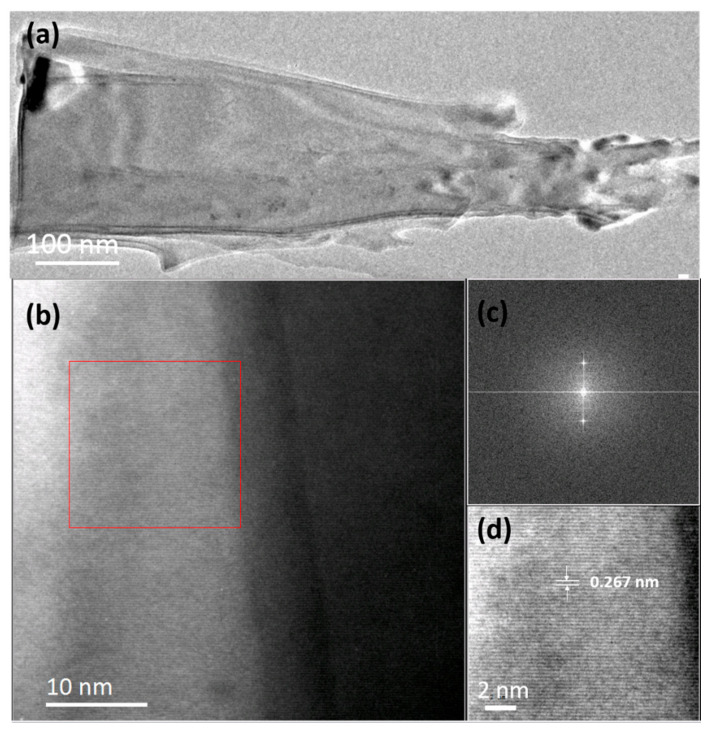
(**a**) Typical cross-sectional TEM imagery of a post-etched nanoarray. (**b**) HRTEM image (scale bar: 10 nm) and (**c**) corresponding FFT (Image J) of the nanostructure within the cone-shaped SiC nanoarrays of a sample etching for 20 min. (**d**) HRTEM image shows the lattice spacing in the red frame area of (**b**). (Scale bar: 2 nm).

TEM samples were prepared by detaching the as-fabricated SiC nanoarrays from the remaining SiC wafer substrate by subjecting them to an ultrasonic treatment for 30 min in absolute ethanol. Figure 4a shows a typical TEM image of a single nanocone under low magnification. Individual SiC nanocones appear to have rough surfaces though retaining very sharp tips with a radius of curvature of approximately 30 nm. Given the relatively low energy density associated with TEM sample preparation, we attribute this surface roughness not to the US treatment but to the RIE process. Figure 4b shows a typical HR-TEM image recorded from the highlighted area in Figure 4a. Note the single-crystalline nature of the as-synthesized nanostructure. Figure 4c shows the corresponding fast Fourier transformation (FFT), which we find is near identical over the entire measured cone, highlighting the crystallographic uniformity of the emitter. The measured *d* spacing of 0.267 nm between two neighboring lattice fringes corresponds to the plane distance of crystalline 4H-SiC, [35] supporting our claims that the etching of the nanoarray is principally along the [10$\bar{1}$0] direction.

**Figure 5:**
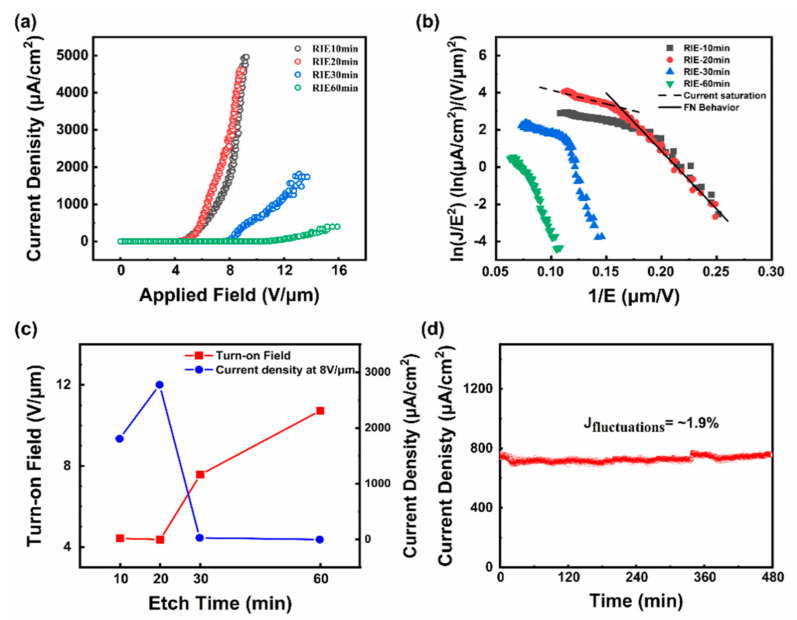
(**a**) Typical current density (J)- electric field (E) curve of the SiC nanoarrays fabricated as a function of etching time with the anode-cathode separation distance fixed at 300 μm. (**b**) The corresponding Fowler–Nordheim (F–N) plots with different etching times. (**c**) The E_to_ and current density applied 8 V/μm of nanoarrays in different etching times. (**d**) The current emission stabilities of SiC nanoarrays at an etching time of 20 min over 8 h.

The FE characteristics of the fabricated SiC nanoarrays were investigated. The current emission density (J) as a function of applied electric field (E) is shown in Figure 5a. In order to study the effect of etching time on the FE performance, the J-E curves of different etching times (10 min, 20 min, 30 min, and 60 min) were measured. Figure 5c shows the extracted turn-on electric field (defined elsewhere [36]) E_to_ with etching time. As the etching time increases, E_to_ increases and J (under a constant field of 8 V/µm) tends to decrease. As the etch time increases, finer nanostructures, due to their energetically preferential geometry, tend to localize the etching plasma, and as a result, they are readily etched away, normalizing the surface, with the diameters of remaining nanostructures gradually become thicker, resulting in the lower field enhancement effect and fewer nanostructures available for effective electron emission. In the context of the FE measurements, we found an optimal etching time to be of the order of 20 min.

Although less extensive than the family of nanocarbons, there have been various theoretical and experimental research projects conducted on FE for a variety of semiconducting materials since the 1960s [37]. Generally, the FE of n-type high-resistance and p-type semiconductors shows non-linear Fowler–Nordheim (F–N) behavior [27,38,39,40,41]. Such J-E profiles can be broadly divided into three regions. As a function of increasing field strength, these include: (1) standard F–N or zero current approximation; (2) saturation region, in which emission is limited by an insufficient supply of carriers due to a p-n junction inverse bias; and (3) rapid increase in emission current as the field penetration becomes sufficient for impact ionization in the space-charge region allowing the carrier density to increase progressively [38]. Others have reported on the electron emission from SiC nanoarrays [41,42,43], noting F–N-like emission where [44],

(1) $$J=\left(\frac{{A\beta }^{2}E^{2}}{\Phi }\right)e^{-{B\Phi }^{\frac{3}{2}}{\left(\beta E\right)}^{-1}}$$

**Figure A3:**
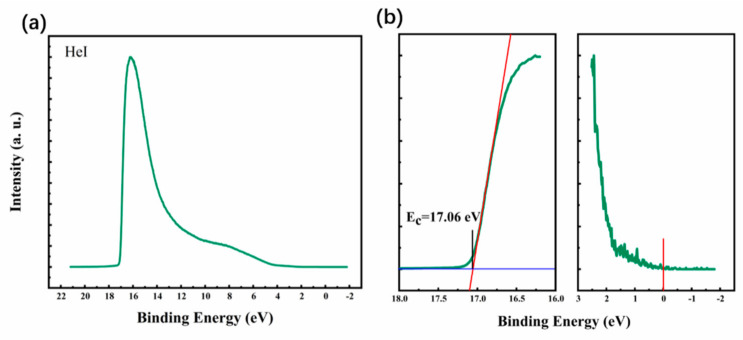
(**a**) Global spectrum of ultraviolet photoelectron spectroscopy. Left and right panel of (**b**) shows the secondary electron cut-off and the Fermi level regions, respectively, to determine the work function (WF).

Here J is current emission density, E is applied field, Φ is the work function of emitting material, A = 1.54 × 10^−6^ A eV V^−2^, B = 6.83 × 10^3^ eV^−3/2^ V μm^−1^ and β is the field enhancement factor. As a wide bandgap semiconductor (E_g_ = 3.23 eV), the FE behavior of SiC is significantly different from that of metallic materials, which can be seen from the observed non-linear F–N characteristics (Figure 5b). These can be divided into F–N behavior and an approximate saturation region, which are respectively linearly fitted (Figure 5b). The slope of F–N behavior plots can be represented by k = −6830 Φ^3/2^/β, thereby allowing β extraction. The k-value depends on the linear slope of the F–N plots and Φ value. Figure A3a shows UPS spectra, and the left/right panel of Figure A3b shows the secondary electron cut-off and the Fermi level regions, respectively. WF is determined from the secondary electron cut-off as Φ = hν–∣E_cut-off_ − E_F_∣, where hν, E_cut-off_ and E_F_ are the photon energy of excitation light (He I discharge lamp, 21.2 eV), the secondary electron cut-off energy, and Fermi level, respectively. The SiC WF was empirically found to be 4.2 (±0.1) eV. Based on this, the corresponding β-values were 1190 (10 min), 1260 (20 min), 418 (30 min), and 483 (60 min). We noticed that the β-value of 30 min is slightly lower than that of 60 min, which is attributed to the reduction of the electrostatic field screening effect caused by the decrease of the emitter density. Compared with bulk materials, due to the high aspect ratio of one-dimensional nanostructures, their FE properties are often significantly improved through the engineering of high aspect ratio one-dimensionality. However, large surface area, low mass, and low melting points make many such high aspect ratio nanostructures vulnerable to chemical and physical erosion and structural damage. Therefore, the stability of current emission is undoubtedly important and remains a challenge in the production of 1D nanomaterial field emitters.

The current emission stability of the as-fabricated field emitters is shown in Figure 5d, which shows the time-dependent emission current over 8 h (RIE-20 min). Measurements were acquired every second at 5 × 10^−6^ Pa and an emission current of 730 μA. The 20 min RIE samples tended to show high emission stability. The emission stability, calculated by,

(2) $$J_{i,\;\mathrm{fluctuation}}=\sqrt{{\left(I_{i}-\;\overset{-}{I}\right)}^{2}}/\;\overset{-}{I}$$

where $\;\overset{-}{I}$ is the average emission current and $I_{i}$ is the current at any moment, stayed within 1.9 ± 1% of the average value of $J_{i,\;\mathrm{fluctuation}}$ across the entire measurement period. We attributed such excellent emission stability mainly to the known high physical and chemical stability of SiC and high dielectric breakdown field strength, high-temperature resistance, and thermal conductivity [2]. These results confirm that SiC nanoarrays etched by RIE could be an excellent candidate for high-power [10,11] stable field emitters, and this preparation method performs a viable means of producing such field emitters.

## 4. Conclusions

Here we have successfully prepared SiC nanoarrays with a highly vertical orientation by adjusting the RIE etching parameters, providing a new low-cost approach for the preparation of nanostructured field emitters. The SiC nanoarrays are spatially dense (~10^7^ mm^−2^), adopt conical morphologies, offer sharp tips (~30 nm) and show high local field enhancement effects (β = 1260). The FE test results show that the turn-on field of the nanoarray is about 4.3 V/μm, indicating competitive FE performance. The long-term current emission stability shows that the 8 h fluctuation is only 1.9 ± 1%, which has high stability and is expected to be used in various applications such as traveling wave tubes and flat-panel displays.

## Data Availability

The data presented in this study are available on request from the corresponding author.
